# Supplementary figures and images for: Multiple Perspectives Reveal the Role of DNA Damage Repair Genes in the Molecular Classification and Prognosis of Pancreatic Adenocarcinoma
Source: Int J Mol Sci. 2022 Sep 6;23(18):10231. doi: 10.3390/ijms231810231 (PMC9499455; doi:10.3390/ijms231810231)

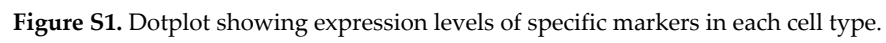

**Figure S1.** Dotplot showing expression levels of specific markers in each cell type.

Supplement: Supplementary file 1 [file ijms-23-10231-s001.zip › Supplementary Figure S1.pdf]
